# Biomechanical investigation of pelvic stability in developmental dysplasia of the hip: unilateral salter osteotomy versus one-stage bilateral salter osteotomy

**DOI:** 10.1186/s13018-020-01683-w

**Published:** 2020-05-11

**Authors:** Lang Li, Xiaodong Yang, Bo Song, Jun Jiang, Lei Yang, Xueyang Tang

**Affiliations:** 1 Department of Pediatric Surgery, West China Hospital, Sichuan University, Chengdu, 610041 Sichuan China; 2 Department of Pediatric Orthopaedic Ward 1, Children’s Hospital of Chongqing Medical University, Chongqing, 400014 China

**Keywords:** Developmental dysplasia of the hip, Salter osteotomy, One-stage, Unilateral

## Abstract

**Background:**

Developmental dysplasia of the hip (DDH) is a common disease in infants and children, and the treatment of bilateral DDH remains controversial. This study aimed to evaluate the stability of one-stage bilateral Salter pelvic osteotomy for bilateral DDH in patients of walking age.

**Methods:**

In total, nine child cadavers aged 2–6 years were included. A universal mechanical testing machine was used for stability test. We performed two different surgical procedures on the specimens: nine child cadavers underwent unilateral Salter pelvic osteotomy, and six child cadavers were randomly selected to undergo Salter pelvic osteotomy again to simulate one-stage bilateral Salter pelvic osteotomy. The stability of the bilateral sacroiliac joints, local stability of the operation area, ultimate load test, and axial stiffness were evaluated.

**Results:**

Both unilateral and bilateral Salter osteotomy could destroy the integrity of the pelvic ring and increase the risk of pelvic instability. In this study, compared with unilateral Salter osteotomy, bilateral Salter osteotomy had similar pelvic stability, and there was no significant difference between unilateral and bilateral Salter osteotomy in sacroiliac joint stability (*p* > 0.05), local stability (*p* = 0.763), ultimate load (*p* = 0.328), and axial stiffness (*p* = 0.480).

**Conclusions:**

One-stage bilateral Salter pelvic osteotomy as a potential surgical method is viable and stable for children with bilateral DDH.

## Introduction

Developmental dysplasia of the hip (DDH) is a common disease in infants and children, and is based on congenital acetabular dysplasia. The incidence of DDH on the left side is 60%, 20% on the right side, and 20% bilaterally [1]. Many infants and children do not receive conservative treatment timeously which leads to abnormal gait/osteoarthritis. As a result, DDH is very common in pediatric orthopedics in developing and low-income countries [2]. Acetabular dysplasia often worsens if the diagnosis is delayed and can cause varying degrees of hip dislocation requiring different treatments for DDH [3–5]. Patients who are diagnosed early can be successfully treated with conservative methods such as a Pavlik harness, but surgical treatment becomes inevitable for most patients with a delayed diagnosis [6].

In 1961, Salter first described open reduction and pelvic osteotomy for DDH. Salter osteotomy has subsequently become a popular treatment option for walking-age children with DDH [7]. Salter pelvic osteotomy changes the direction of the acetabulum by cutting the ilium completely and using the pubic symphysis as a hinge to rotate the distal end of the osteotomy bone. This increases accommodation of the dislocated femoral head to achieve central reduction [7, 8]. Salter pelvic osteotomy involves cutting the ilium followed by fixation with Kirschner wires; this can affect the stability of the pelvis, therefore some surgeons suggest that patients with bilateral DDH should receive successive Salter osteotomy because of the high risk of a poor Severin grading [9]. And Salter also thought one-stage bilateral Salter pelvic osteotomy to be contraindicated and recommended that the osteotomy on the second hip be performed two weeks after the first hip. Salter thought one-stage bilateral Salter pelvic osteotomy could result with loss of fixation [2]. However, some studies have also reported that one-stage bilateral pelvic osteotomy is reliable and economical [10–12]. One-stage bilateral osteotomy was viable and may be even better acetabular index than that of a unilateral osteotomy. However, there are currently no biomechanical experiment to explore and verify the pelvic stability of one-stage bilateral Salter pelvic osteotomy.

We are of the opinion that one-stage bilateral pelvic osteotomy with ilia cut bilaterally, will result in the same load on the left and right of the pelvic ring, thereby maintaining balance, and will not increase the risk of pelvic instability. In this study, we prepared pelvic specimens to evaluate the stability of one-stage bilateral Salter pelvic osteotomy in children aged 2 to 6 years.

## Materials and methods

Pelvic specimens from four male and five female child cadavers aged 2–6years were obtained from the Department of Anatomy, West China of Medicine, Sichuan University. Approval was obtained from the Sichuan Province in accordance with the law for the protection of cadavers sampling of the Chinese Government.

### Sample preparation

**Fig. 1:**
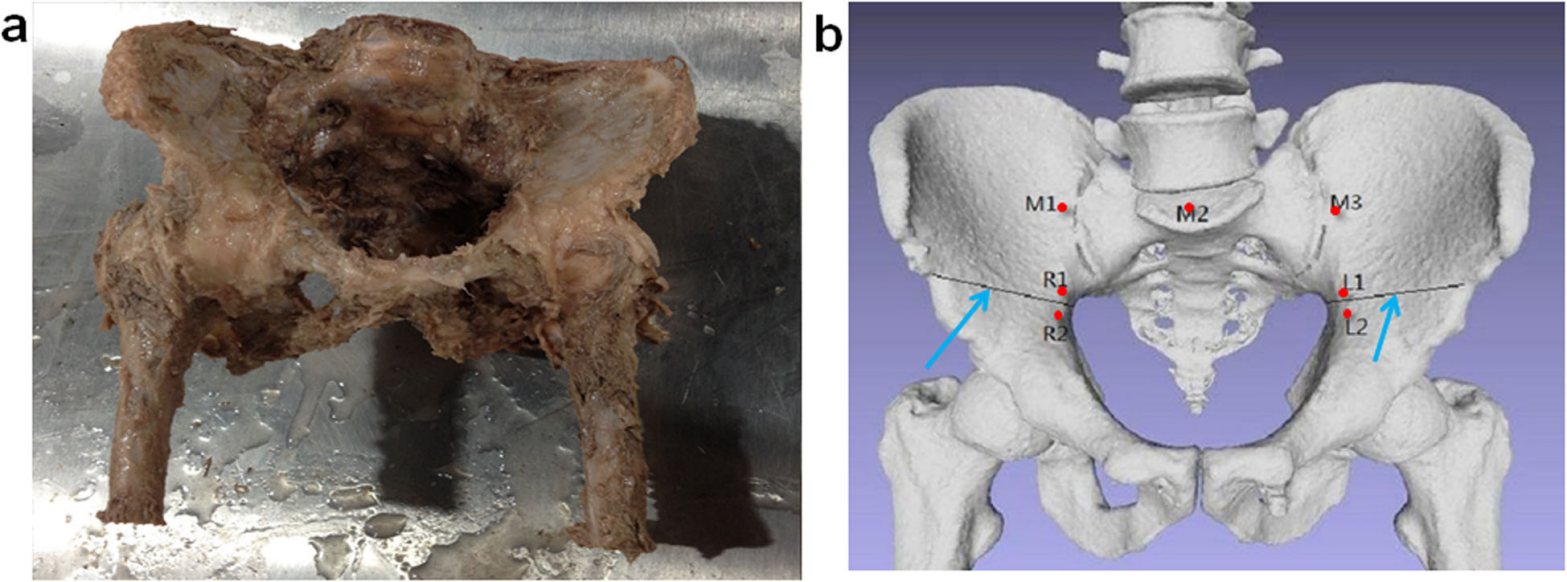
Intact pelvis and marked point. **a** The intact pelvis before treated with different operation. **b** The marked point of M1, M2, M3, L1, L2, R1, and R2. M1 and M3 were marked on the iliac side of the sacroiliac joint, respectively, and M2 was marked at the same level on the sacrum. L1(R1) and L2(R2) were marked on both sides of Salter osteotomy, respectively. The blue arrow refers to the broken end of the osteotomy

**Fig. 2:**
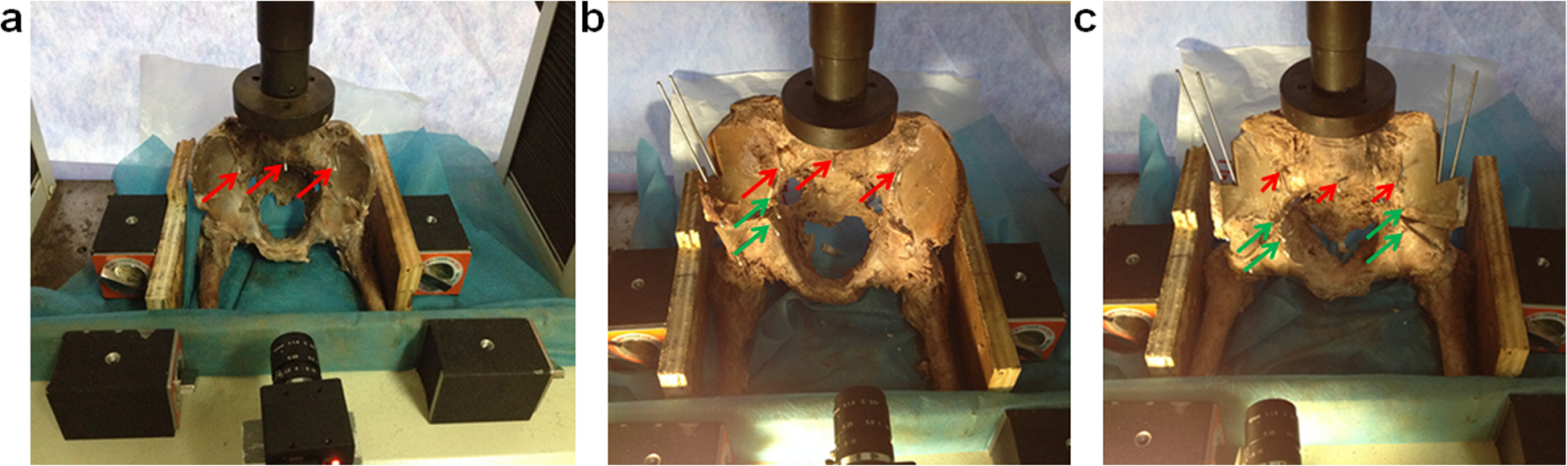
Different osteotomy for pelvis and the displacement of marked point was acquired by sophisticated camera. **a** The intact pelvis. **b** The unilateral salter pelvic osteotomy. **c** One-stage bilateral Salter pelvic osteotomy. The red arrow refers to M1 or M2 or M3, and green arrow refers to L1(R1) and L2(R2)

Nine child cadavers including five girls were unfrozen 1 day prior to experimrnt, and the pelvic specimens were dissected by a professional surgeon. The pelvic specimens were complete from the fifth lumbar vertebra to 10 cm of the proximal femur. The sacroiliac joint, hip joint, pubic symphysis, and related ligament were protected (Fig. 1a). Nine pelvic specimens were prepared for pelvic stability examination as follows:

The pelvis was fixed in a standing position, and stability was tested using a universal mechanical testing machine (AG-IS, Shimadzu, Japan). First, M1 and M3 were marked on the iliac side of the sacroiliac joint, respectively, and M2 was marked at the same level on the sacrum (Fig. 1b). The force added to the pelvis on the top of the fifth lumbar vertebra was from 0 to 400 N with a speed of 10 N/S (Fig. 2a). Images were acquired every four seconds using a sophisticated camera (JHSM1400f, China), and displacements of M1–M3 were converted and processed by a computer. The principle of image displacement recognition method is as follows: the coordinate origin is directly above the M2 point; the *X*-axis direction represents the horizontal displacement to the right, and the *Y*-axis direction represents the vertical displacement downwards. The positions of M1(x1, y1), M2(x2, y2), and M3(x3, y3) were obtained.

### Unilateral Salter pelvic osteotomy

Unilateral Salter pelvic osteotomy was performed on nine pelvic specimens by a professional surgeon; left/right ilium was chosen randomly with random numbers generated by computer. The implanted bone graft was taken from the ipsilateral ilium as an isosceles triangle and was the same basic size for all pelvic specimens (base is 2 cm, apex angle is 30°). Two Kirschner wires were used to fix the implanted bone graft. After Salter osteotomy was performed, four points (L1, L2, L3, and L4) were marked (Fig. 1b). A force from 0 to 400 N with a speed of 10 N/S was added to the pelvis on the top of the fifth lumbar vertebra. The displacements of M1, M2, M3, L1, L2, R1, and R2 were recorded and processed using the method described above (JHSM1400f, China) (Fig. 2b).

### Bilateral Salter pelvic osteotomy

Six pelvic specimens that underwent unilateral Salter pelvic osteotomy were chosen randomly with a computer random number to receive another Salter pelvic osteotomy to simulate bilateral Salter pelvic osteotomy. The osteotomy was performed by a same professional surgeon as mentioned above. A force from 0 to 400 N with a speed of 10 N/S was added to the pelvis. Seven points (M1, M2, M3, L1, L2, R1 and R2) were marked and all displacements were recorded (Fig. 2c).

### Axial stiffness and ultimate load test

The vertical displacement of M2 (VD-M2) is an approximate representation of the displacement of the pelvic ring. In this study, we used 400 N/VD-M2 to determine axial stiffness. After the force test from 0 N to 400 N, we performed an ultimate load test to evaluate the stability of the pelvic ring after different surgeries. A force from 0 N was added to the pelvis on the top of the fifth lumbar vertebra with a speed 10 N/S. Ultimate load was defined as the force that damaged the pelvis and caused the pelvic ring to become unstable.

### Statistical analysis

All measurement data were presented as the mean and standard deviation. The unpaired two-tailed t-test or one-way ANOVA using SPSS (version 19.0, USA) were used to determine whether there were any statistically significant differences between the means of the different treatment groups. Differences were considered significant when *p* values < 0.05.

## Results

### Stability of bilateral sacroiliac joints

**Fig. 3:**
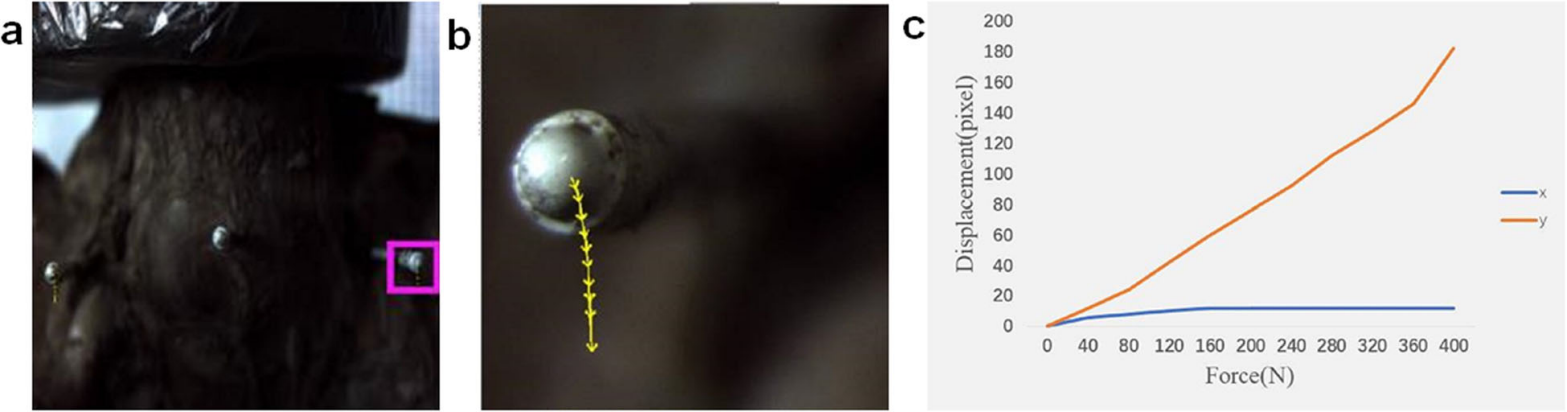
The movement locus of marked point when a force added to the pelvis on the top of the fifth lumber from 0 to 400 N. **a** The marked point in pink rectangle were analyzed. **b** The yellow line represents the movement locus of marked point in pink rectangle. **c** The blue and red line represents displacement of *X*-axis direction and *Y*-axis direction respectively when the force added from 0 N to 400 N

**Fig. 4:**
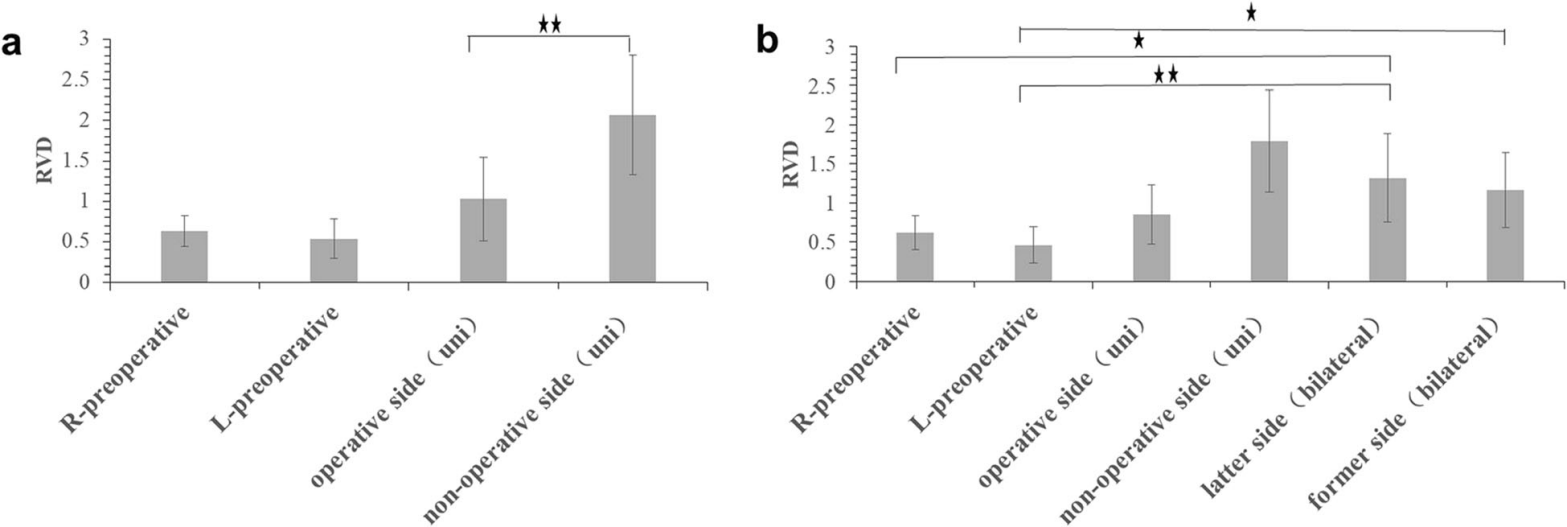
Assessment of stability of bilateral sacroiliac joints. **a** Stability comparison of intact pelvis and pelvis treated with unilateral salter pelvic osteotomy. **b** Stability comparison among intact pelvis, pelvis treated with unilateral Salter pelvic osteotomy and one-stage bilateral Salter pelvic osteotomy (^⋆^*p* < 0.05, ^⋆⋆^*p* < 0.01)

Nine pelvic specimens were subjected to a force from 0 N to 400 N, and displacement was measured using a sophisticated camera. Baseline displacement of M1, M2, and M3 was obtained prior to surgery. The results showed that displacement was represented mainly on the *Y*-axis (Fig. 3a–c). As the pelvic ring is fixed in the horizontal position, only vertical displacement is discussed in the following experiment. We used the relative vertical displacement (RVD) of M1 and M2 to represent the stability of the right sacroiliac joint, and the RVD of M2 and M3 to represent the stability of the left sacroiliac joint. The RVD of the right and left sides, before Salter pelvic osteotomy, were denoted by D1 and D2, respectively. D1 and D2 of the nine pelvic specimens were 0.63 ± 0.19 mm and 0.54 ± 0.24 mm, respectively. There was no significant difference between D1 and D2 (*p* = 0.967). Unilateral Salter pelvic osteotomy was applied to nine pelvic specimens, and the RVD for the left and right sides was recorded. D3 and D4 represent the RVD of the operated and non-operated sides, respectively. D3 was 1.03 ± 0.52 mm, which was significantly lower than D4 (2.07 ± 0.74 mm, *p* < 0.001). D3 was not significantly different compared with D1 (*p* = 0.192) and D2 (*p* = 0.081) whereas D4 was significantly higher than both D1 and D2 (*p* < 0.001) (Fig. 4a). To assess whether one-stage bilateral Salter pelvic osteotomy affects pelvic stability, we chose six pelvic specimens for further (bilateral) osteotomy. D5 represents the RVD of the former (initial) osteotomy side and D6 represents the RVD of the latter (subsequent) osteotomy side. The value of D5 was 1.32 ± 0.57 mm, which is not significantly different from D6 at a value of 1.16 ± 0.48 mm (*p* = 0.997). The values of D1, D2, D3, and D4 of the six pelvic specimens were 0.62 ± 0.22 mm, 0.46 ± 0.23 mm, 0.85 ± 0.38 mm, and 1.79 ± 0.65 mm, respectively. D5 was significantly higher than D1 and D2 (*p* < 0.05). D6 was also higher than D1 and D2 and the difference between D6 and D2 was significant. Both D5 and D6 were higher than D3 and lower than D4, but the differences were not significant (*p* > 0.05) (Fig. 4b).

### Local stability of operation area

**Fig. 5:**
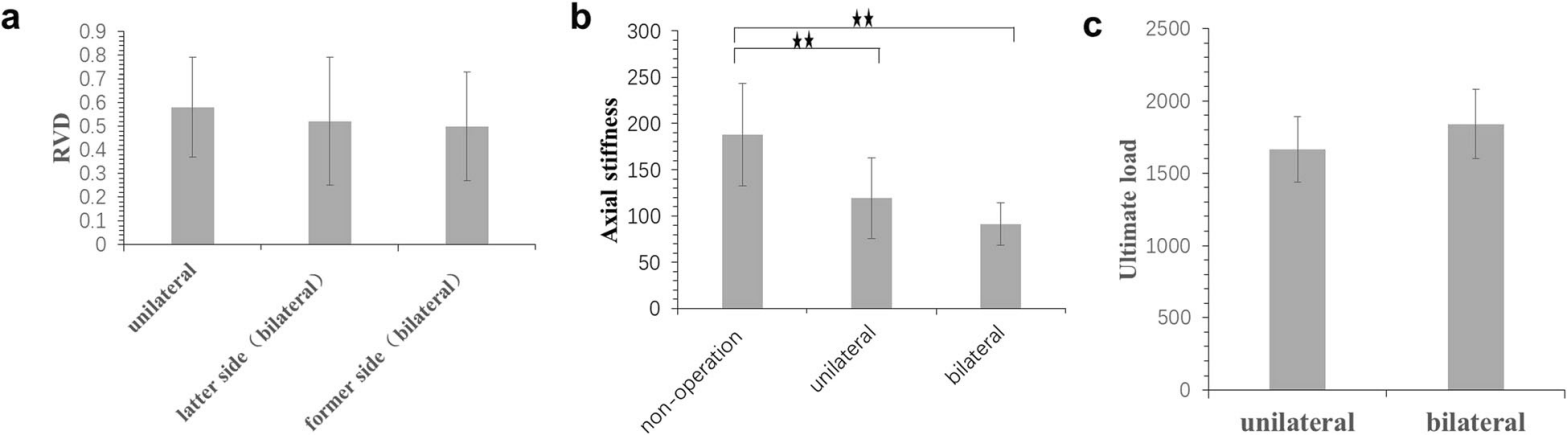
Assessment of stability of local stability of operation area, ultimate load test and axial stiffness. **a** The RVD of local stability of operation area for different operation sites. **b** comparison of axial stiffness among non-operation group, unilateral salter pelvic osteotomy group and one-stage bilateral Salter innominate osteotomy group. **c** Comparison of ultimate load for unilateral salter pelvic osteotomy and one-stage bilateral Salter innominate osteotomy (^⋆⋆^*p* < 0.01)

The RVD of L1 to L2, and R1 to R2 represent the local stability of the operation area. D7 and D8 represent the RVD of the former (initial) operation side and the latter (subsequent) operation side, respectively. Unilateral Salter pelvic osteotomy was applied to nine pelvic specimens and the value of D7 (unilateral) was 0.58 ± 0.21 mm. The one-stage bilateral Salter pelvic osteotomy was applied to six pelvic specimens. The values of D7 (bilateral) and D8 (bilateral) of these six pelvic specimens were 0.50 ± 0.23 mm, and 0.52 ± 0.27 mm, respectively. There was no significant difference between D7 (unilateral), D7 (bilateral), and D8 (bilateral) (*p* = 0.763) (Fig. 5a).

### Axial stiffness assessment

The baseline axial stiffness was 188.11 ± 55.41 N/mm before Salter osteotomy. After unilateral Salter pelvic osteotomy, the axial stiffness was lower (119.06 ± 43.47 N/mm). For one-stage bilateral Salter pelvic osteotomy specimens, the axial stiffness was 91.30 ± 22.42 N/mm, which was lower than both the unilateral Salter pelvic osteotomy group and the non-operation group. There was a significant difference in axial stiffness between the non-operation group and both the unilateral Salter pelvic osteotomy group (*p* = 0.010), and the one-stage bilateral Salter pelvic osteotomy group (*p* = 0.001). However, there was no significant difference between the unilateral and one-stage bilateral Salter pelvic osteotomy groups (*p* = 0.480) (Fig. 5b).

### Ultimate load test

The ultimate load test was applied to the unilateral and bilateral Salter pelvic osteotomy specimens. For the unilateral Salter pelvic osteotomy specimens, the ultimate load was 1664.27 ± 226.26 N and 1840.83 ± 241.55 N for the six one-stage bilateral Salter pelvic osteotomy specimens. There was no significant difference in the ultimate load between the two different treatment groups (*p* = 0.328) (Fig. 5c). All results of our research were also shown in Supplementary Table S1.

## Discussion

DDH is a progressive disease with varying severity in children. The stability of the pelvic ring is mainly maintained by the posterior sacroiliac joint, the anterior pubic symphysis, and the surrounding ligaments, which tightly bind all bone blocks together to form the pelvic ring [13, 14]. In this study, nine child cadaver specimens were subjected to unilateral and six of those were subjected to one-stage bilateral Salter pelvic osteotomy. We found that one-stage bilateral Salter osteotomy had a similar effect on sacroiliac joint stability, local stability, axial stiffness, and ultimate load as unilateral Salter pelvic osteotomy.

The sacroiliac joint, and especially the surrounding ligaments, play an important role in the stability of the posterior pelvic ring. Salter pelvic osteotomy damaged the pelvic ring, but we found that the sacroiliac joint at the operation side after unilateral Salter pelvic osteotomy was more stable than at the non-operation side. This may be caused by a bilateral asymmetrical force. In Barnes’s study, the researchers found that unilateral Salter pelvic osteotomy could lead to hip dysplasia on the healthy side [15]. We think that the pelvic stress on the operation side was transferred to the contralateral pelvic ring, as force hinge, carrying more loads which affected the stability of the non-operation side. However, one-stage bilateral Salter pelvic osteotomy cuts both the left and right ilium and both sides are fixed with the same treatment. This could lead to two hinge points making bilateral sacroiliac joint stress a symmetrical force.

Salter pelvic osteotomy cuts the ilium and is fixed with Kirschner wires. This study tested whether one-stage bilateral Salter pelvic osteotomy increases the risk of local stability. We found that the RVD of local stability for one-stage bilateral Salter pelvic osteotomy was between 0.50 and 0.52 mm, which is lower than that for unilateral Salter pelvic osteotomy. Moreover, we considered that one-stage bilateral Salter pelvic osteotomy created a bilateral symmetrical force on both the left and right pelvic ring.

The axial stiffness of the pelvis reflects the displacement of the pelvis when subjected to axial pressure [16]. The greater the axial stiffness, the stronger the pelvic compressive capacity. We found that both unilateral and one-stage bilateral Salter pelvic osteotomy decreased the axial stiffness of the pelvis. In addition, this study showed axial stiffness of one-stage bilateral pelvic osteotomy (91.30 N/mm) to be lower than that of unilateral Salter pelvic osteotomy (119.06 N/mm) but the difference was not significant. The ultimate load reflects the stability of the whole pelvic ring, and we found that one-stage bilateral Salter pelvic osteotomy did not decrease ultimate load, which verified the stability of one-stage bilateral Salter pelvic osteotomy. Interestingly, the ultimate load test showed that, regardless of whether unilateral or bilateral pelvic Salter osteotomy was performed, the pelvic ring was first destroyed at the sacroiliac joint, and not at the Kirschner wire fixation site. According to the current weight estimation formula “Weight (kg) = 2(age +4)” [17], the ultimate load of two Salter pelvic osteotomies is strong enough to support at least eight times the body weight of a child aged 6 years. All the results support the theory that one-stage bilateral pelvic Salter osteotomy is vertically stable. However, due to the preciousness of the cadaver specimens, the study did not test the ultimate load of a normal pelvic ring, so no comparison between a normal pelvic ring and a bilateral one-stage osteotomy pelvic ring was done in this study.

This study was the first to confirm the stability of one-stage bilateral Salter pelvic osteotomy. Some surgeons have tried one-stage surgery for DDH disease and have followed up on efficacy. In Ochoa’s study, 45 children suffering from congenital dislocation of the hip or acetabular dysplasia were assigned to one-stage bilateral Salter pelvic osteotomy (15 children) or successive Salter osteotomy (30 children). One-stage osteotomy was shown to be viable and had a better acetabular index and may be even better than that of a unilateral osteotomy [18]. In another study [19], a one-stage operation (using a Pemberton osteotomy for one hip and a Salter osteotomy for the other hip) was more economical and allowed more rapid recovery than a two-stage procedure comprising consecutive operations. In addition to Salter osteotomy, the stability of a one-stage Pemberton’s pericapsular osteotomy, which also damages the pelvic ring, has been verified. In Zorer’s study, 20 patients underwent a one-stage bilateral Pemberton’s pericapsular osteotomy, and the results showed significant advantages over 2 separate consecutive interventions [20]. Another study by Agus’ team evaluated the clinical outcome of 12 children (24 hips) who treated with one-stage bilateral Salter pelvic osteotomy and compared with 12 patients (12 hips) who received unilateral Salter pelvic osteotomy, and found one-stage bilateral Salter pelvic osteotomy did not increase the mortality and postoperative complications [21]. And only the blood transfusion volume was higher in one-stage bilateral Salter pelvic osteotomy group than that of unilateral osteotomy group (170 cc vs 100 cc), which may be because bilateral osteotomy group needs longer operation time.

Although the stability of a one-stage bilateral Salter pelvic osteotomy was verified in this study, there were some limitations. First, there were a limited number of cadaveric pelvic specimens, and only six underwent one-stage bilateral Salter pelvic osteotomy. Second, the cadaver specimens belong to kids without any DDH, our model could not completely simulate live patients. The weight of the upper body is mainly transmitted to the pelvic ring through the bones, especially the spine, but some tissues such as skin, fascia, and muscle of psoas, and gluteals also transmit pressure. Third, according to our clinical experience, children who undergo one-stage bilateral Salter pelvic osteotomy receive plaster fixation and are allowed to start some exercises in bed before pelvic osteotomy healing, whereas the specimens in the study were static and the early postoperative activities of children could not be simulated. Fourth, in Salter osteotomy, the fulcrum point is the pubic symphysis, we evaluated the RVD of the surgical site, but did not evaluate the anterior and lateral displacement of the acetabulum on the contralateral side. In addition, the pubic symphysis, as the force hinge of Salter osteotomy, was very meaningful to explore the stability difference between unilateral and bilateral salter osteotomy. In future research, we need to further explore and improve these deficiencies. Despite these limitations, some important structures were protected and showed that one-stage bilateral Salter pelvic osteotomy is viable and stability for bilateral DDH.

In summary, this study was based on the pelvis of children aged 2 to 6 years and simulated one-stage bilateral Salter pelvic osteotomy. Compared with unilateral Salter pelvic osteotomy, one-stage bilateral Salter pelvic osteotomy is viable and stability from the aspect of sacroiliac joint stability, local stability, ultimate load, and axial stiffness. However, this finding was based on experimental research. Clinical treatment of patients is more complicated and is affected by many factors, such as the physical condition of the patient, hospital infrastructure, anesthesia level, doctor’s experience, and patient compliance. We must carefully choose treatment options. In addition, a larger number of specimens and long-term clinical follow-up are necessary in future.

## Supplementary information

**Additional file 1:** Tables S1 Table the detail results of sacroiliac joint stability, local stability, ultimate load, and axial stiffness.

## Data Availability

The datasets used and/or analyzed during the current study are available from the corresponding author on reasonable request.

## Abbreviations

DDH: Developmental dysplasia of the hip
RVD: Relative vertical displacement
