# Supplementary material for: Biomechanical investigation of pelvic stability in developmental dysplasia of the hip: unilateral salter osteotomy versus one-stage bilateral salter osteotomy
Source: J Orthop Surg Res. 2020 May 11;15:169. doi: 10.1186/s13018-020-01683-w (PMC7216724; doi:10.1186/s13018-020-01683-w)
Supplement: Supplementary file 1 — Additional file 1: Tables S1 Table the detail results of sacroiliac joint stability, local stability, ultimate load, and axial stiffness. [file 13018_2020_1683_MOESM1_ESM.docx]

| S1 Table the detail results of sacroiliac joint stability, local stability, ultimate load, and axial stiffness | | | | |
| --- | --- | --- | --- | --- |
| **Stability of bilateral sacroiliac joints(preoperative vs unilateral osteotomy)** | | | | |
|  | Symbol | sample size（N） | RVD（mm） | P |
| preoperative（Right） | D1 | 9 | 0.63±0.19 |  |
| preoperative（left） | D2 | 9 | 0.54±0.24 | D3 vs D4, P<0.001 |
| Unilateral osteotomy（operation side） | D3 | 9 | 1.03±0.52 | D1 vs D4, P<0.001 |
| Unilateral osteotomy（non-operation side） | D4 | 9 | 2.07±0.74 | D2 vs D4, P<0.001 |
|  |  |  |  |  |
| **Stability of bilateral sacroiliac joints(preoperative vs unilateral vs bilateral)** | | | | |
|  |  | sample size（N） | RVD（mm） | P |
| preoperative（Right） | D1 | 6 | 0.62±0.22 |  |
| preoperative（left） | D2 | 6 | 0.46±0.23 | D5 vs D2, P=0.007 |
| Unilateral osteotomy（operation side） | D3 | 6 | 0.85±0.38 | D5 vs D1, P=0.037 |
| Unilateral osteotomy（non-operation side） | D4 | 6 | 1.79±0.65 | D6 vs D2, P=0.037 |
| Bilateral osteotomy（latter side） | D5 | 6 | 1.32±0.57 |  |
| Bilateral osteotomy（former side） | D6 | 6 | 1.16±0.48 |  |
|  |  |  |  |  |
| **local stability of the operation area (unilateral vs bilateral)** | | | | |
|  |  | sample size（N） | RVD（mm） | P |
| Unilateral osteotomy（former side） | D7'' | 9 | 0.58±0.21 |  |
| Bilateral osteotomy（former side） | D7 | 6 | 0.52±0.27 | 0.763 |
| Bilateral osteotomy（latter side） | D8 | 6 | 0.50±0.23 |  |
|  |  |  |  |  |
| **ultimate load(unilateral vs bilateral)** | | | | |
|  |  | sample size（N） | RVD（mm） | P |
| Unilateral osteotomy |  | 3 | 1664.27±226.26 | 0.328 |
| Bilateral osteotomy |  | 6 | 1840.83±241.55 |  |
|  |  |  |  |  |
| **axial stiffness(unilateral vs bilateral)** | | | | |
|  |  | sample size（N） | RVD（mm） | P |
| preoperative | C1 | 9 | 188.1152±55.40831 | C2 vs C3, P=0.480 |
| Unilateral osteotomy | C2 | 9 | 119.0642±43.47126 | C1 vs C2, P=0.010 |
| Bilateral osteotomy | C3 | 6 | 91.3020±22.42249 | C1 vs C3, P=0.001 |
|  |  |  |  |  |
